# Dialdehyde Starch as a Cross-Linking Agent Modifying Fish Collagen Film Properties

**DOI:** 10.3390/ma17071475

**Published:** 2024-03-23

**Authors:** Patrycja Brudzyńska, Karolina Kulka-Kamińska, Łukasz Piwowarski, Katarzyna Lewandowska, Alina Sionkowska

**Affiliations:** 1 Department of Biomaterials and Cosmetic Chemistry, Nicolaus Copernicus University in Torun, Gagarin 7, 87-100 Torun, Poland; patrycja.brudzynska@umk.pl (P.B.); kkulka@doktorant.umk.pl (K.K.-K.); reol@umk.pl (K.L.); 2 SanColl Sp. z o.o., Juliusza Słowackiego 24, 35-060 Rzeszów, Poland; aktivpiwowarski@gmail.com

**Keywords:** collagen, dialdehyde starch, cross-linking, biopolymer film

## Abstract

The aim of this research was the modification of fish collagen films with various amounts of dialdehyde starch (DAS). Film properties were examined before and after the cross-linking process by DAS. Prepared biopolymer materials were characterized by Fourier Transform Infrared Spectroscopy and Atomic Force Microscopy. Moreover, the mechanical, thermal and swelling properties of the films were evaluated and the contact angle was measured. Research has shown that dialdehyde starch applied as a cross-linking agent influences collagen film properties. Mechanical testing indicated a decrease in Young’s Modulus and an increase in breaking force, elongation at break, and tensile strength parameters. Results for contact angle were significantly higher for collagen films cross-linked with DAS; thus, the hydrophilicity of samples decreased. Modified samples presented a lower swelling degree in PBS than native collagen films. However, the highest values for the degree of swelling among the modified specimens were obtained from the 1% DAS samples, which were 717% and 702% for 1% and 2% collagen, respectively. Based on AFM images and roughness values, it was noticed that DAS influenced collagen film surface morphology. The lowest value of Rq was observed for 2%Coll_2%DAS and was approximately 10 nm. Analyzing thermograms for collagen samples, it was observed that pure collagen samples were less thermally stable than cross-linked ones. Dialdehyde starch is a promising cross-linking agent for collagen extracted from fish skin and may increase its applicability.

## 1. Introduction

Among numerous biopolymers, collagen offers many possibilities in biomedical and cosmetic applications and plays an important role in designing wound-dressing materials or scaffolds for bone regeneration. It is a valuable biopolymer in tissue engineering and biomedical materials due to its biodegradability, biocompatibility, non-toxicity, and good mechanical properties of matured collagen [1,2]. This insoluble fibrous protein consists of three chains built up from amino acids such as glycine, proline, and hydroxyproline and forms a helical structure [3,4]. Twenty-nine types of this protein can be distinguished. Collagen, mainly type I, II, and III, is present in human organisms and can be found in skin (type I and type III), bone tissue, tendons, or blood vessels [5,6,7,8,9]. For industrial purposes, usually porcine or bovine collagen is used but a source of collagen rather free from disease transmission can be extracted from marine organisms [4]. Collagen can be obtained from fish waste material such as skin, fins, scales, or bones [10,11]. Marine collagen compared to mammalian collagen is more bioavailable and is characterized by a higher absorption capability; however, its lower temperature of denaturation poses a challenge in designing new materials [12]. Presently, collagen is eagerly modified with selected synthetic and natural additives or blended with other biopolymers [13,14,15,16,17,18]. New cross-linking agents are constantly being researched so that the modification of collagen properties makes it suitable for various purposes. Collagen can be cross-linked physically by temperature or UV irradiation, enzymatically or chemically. Great emphasis is placed on non-cytotoxic cross-linking agents allowing the use of collagen materials for biomedical purposes. Many different substances are widely used for the chemical cross-linking of collagen. Among them, glutaraldehyde, genipine, EDC-NHS (1-Ethyl-3-(3-dimethylaminopropyl) carbodiimide and N-hydroxysuccinimide), and chitosan can be distinguished [19]. Also, tannic acid is suitable for cross-linking processes [20]. Moreover, one of the cross-linking agents can be dialdehyde starch. This compound is obtained by the oxidation of starch, for example, by periodate. Oxidation significantly influences starch properties, especially solubility, swelling degree, and thermal properties. As a result of this process, the formed dialdehyde starch contains reactive aldehyde groups and due to its cross-linking ability, it is a valuable macromolecule in biomedical applications [21]. Many research groups used this environmentally friendly chemical, which is characterized by lower toxicity compared to aldehyde compounds and compatibility with blood [22]. Valipour et al. indicated in their studies that dialdehyde starch addition to collagen and chitosan hydrogels influenced swelling ratio and biodegradability. Prepared hydrogels based on collagen, chitosan, and dialdehyde starch presented appropriate water vapor transmission for wound-healing materials [22]. Dialdehyde starch was also used for cross-linking scaffolds made from collagen, chitosan, and silk fibroin [23] or collagen, chitosan, and hyaluronic acid, resulting in a higher compressive modulus than those obtained with tannic acid [24], improved mechanical properties, density, porosity and biocompatibility [25], lower roughness, lower elasticity, and higher resistance to rupture of samples [26]. Scaffolds consisting of gelatin and chitosan modified with dialdehyde starch were characterized by increased mechanical strength, zeta potential, swelling properties, and porosity [27]. Furthermore, Wang et al. studied the fixation effect of dialdehyde starch on decellularized porcine aortas aiming to replace glutaraldehyde. Modification with dialdehyde starch increases the tensile strength of samples and improves resistance to enzymatic degradation and anti-calcification capability [28]. Dialdehyde starch was also applied to modify biodegradable films and foils obtained from collagen hydrolysate [29,30,31]. Liu et al. investigated the effects of this biopolymer on the calcification of the collagen matrix and the results of their studies indicated that dialdehyde starch can improve the biological and physical properties of collagen [32]. Collagen cryogels were also cross-linked with dialdehyde starch with improved thermal stability in comparison to pure collagen [33].

Based on review of the literature, our hypothesis is as follows: Can we modify fish skin collagen by DAS? The aim of the study was to prepare biopolymer films made of collagen extracted from fish skin and cross-linked by DAS with improved thermal stability and mechanical and swelling properties compared to native collagen. The films were prepared from two concentrations of collagen with various amounts of DAS. Film properties before and after cross-linking were characterized by Fourier Transform Infrared Spectroscopy and Atomic Force Microscopy. Moreover, the mechanical properties of the films were evaluated and contact angle measurements were performed. Moreover, thermogravimetric analysis and swelling analysis were performed.

## 2. Materials and Methods

### 2.1. Solution and Film Preparation

In this research, collagen from fish skin was used. After the extraction of collagen from silver carp fish skin, the specimens were freeze-dried. Collagen was extracted from silver carp fish skin by SanColl Sp. z o.o., Władysławowo, Poland. Freeze-dried collagen was dissolved in 0.1 M acetic acid (Stanlab; Lublin, Poland) at room temperature to prepare collagen solutions of two concentrations: 1% and 2% (*w*/*w*). The process lasted several days, during which solutions were shaken and stirred on a magnetic stirrer (500 rpm). A solution casting method was applied to obtain collagen films (10 × 10 cm polystyrene plate was used). Dialdehyde starch (Biosynth, Bratislava, Slovakia) solutions were prepared in two concentrations, 3 mg/mL and 6 mg/mL, at room temperature. Subsequently, 1, 2, and 5 mL of DAS was added to 30 g of 1 and 2% collagen solutions so that the addition of the cross-linking agent based on the biopolymer content in the sample was 1, 2, and 5%, respectively. Samples were marked as follows: 1%Coll, 1%Coll_1%DAS, 1%Coll_2%DAS, 1%Coll_5%DAS, 2%Coll, 2%Coll_1%DAS, 2%Coll_2%DAS, 2%Coll_5%DAS. After DAS addition, solutions were stirred for 5 min (500 rpm) and left to dry at room temperature. Films obtained from the 1% collagen solution were 0.02 mm thick, while those from the 2% collagen solution were 0.04 mm thick. Photos of prepared collagen films with DAS addition are presented in Table 1.

### 2.2. Mechanical Properties

A mechanical testing machine (Z.05, Zwick and Roell, Ulm, Germany) was applied to determine Young’s Modulus (GPa), elongation at break (%), breaking force (N), and tensile strength (MPa) parameters of collagen film samples with the speed starting position equaling 50 mm/min, the speed of the initial force equaling 5 mm/min, and the initial force 0.1 equaling MPa. All mechanical measurements were performed under the same conditions of temperature and humidity. Data were collected with the TestXpert II 2017 program.

### 2.3. FTIR Spectroscopy

A Nicolet iS10 spectrometer equipped with a diamond ATR accessory (Thermo Fisher Scientific, Waltham, MA, USA) was used to register infrared spectra of prepared films (resolution: 4 cm^−1^, wavenumber range: 400–4000 cm^−1^, 64 scans).

### 2.4. Swelling and Degradation Test

The swelling degree was calculated for obtained collagen films. For each collagen film, five square-shaped samples were prepared, weighing about 0.0050 g, and then placed in 50 mL phosphate-buffered saline (PBS) at 37 °C. The phosphate-buffered solution was prepared from PBS tablets (make up to 500 mL with distilled water). PBS tablets were bought from Life Technologies Limited (UK). Samples, gently dried on paper, were weighted after 1 h, 2 h, 4 h, 8 h, 24 h, 48 h, 72 h, 1 week, and 2 weeks. The following equation was used to calculate the swelling degree:

$$swelling=\frac{\left(m_{t}-m_{0}\right)}{m_{0}}\;\times \;100\%\;\;\;\;\;\;\;\;\;\;\;\;\left[\%\right]$$

*m_t_*—weight of the material after immersion in PBS [g].

*m*_0_—initial weight of the material [g].

### 2.5. Contact Angle

Contact angle measurements were performed using a goniometer with a drop shape analyzer system (DSA 10, Krüss, Germany). About 10 measurements for each film were performed for two liquids, diiodomethane (D) and glycerine (G), at room temperature. Surface free energy was calculated by the Owen–Wendt method.

### 2.6. Atomic Force Microscope

Surface morphology and roughness of collagen films were characterized with images obtained from atomic force microscopy (AFM) by a MultiMode Scanning Probe Microscope Nanoscope IIIa (Digital Instruments Veeco Metrology Group, Santa Barbara, CA, USA) with tapping mode at room temperature. Nanoscope software (v6.11, Bruker Optik, Ettlingen, Germany) was applied to calculate the roughness parameters for the scanned area 5 μm × 5 μm.

### 2.7. Thermogravimetric Analysis

Thermogravimetric analyses were performed with a Jupiter STA 449 F5 thermal analyzer (Netzsch, Selb, Germany) combined with a FT-IR Vertex 70V spectrometer (Bruker Optik, Ettlingen, Germany) in the temperature range from 20 to 600 °C at a heating rate of 20 °C/min in a nitrogen atmosphere.

## 3. Results

### 3.1. FTIR Spectroscopy

**Figure 1:**
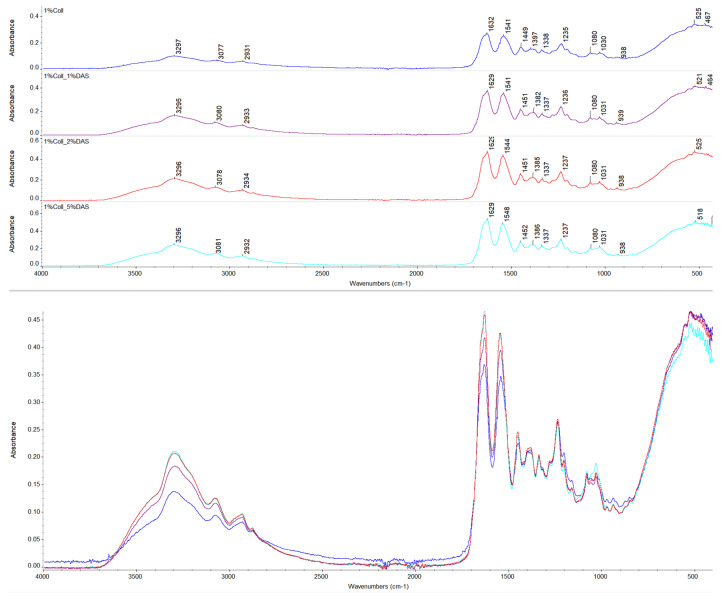
FTIR spectra for collagen films obtained from 1% solution with DAS addition (experiment conditions: resolution = 4 cm^−1^; wavenumber range = 400–4000 cm^−1^; 64 scans).

**Figure 2:**
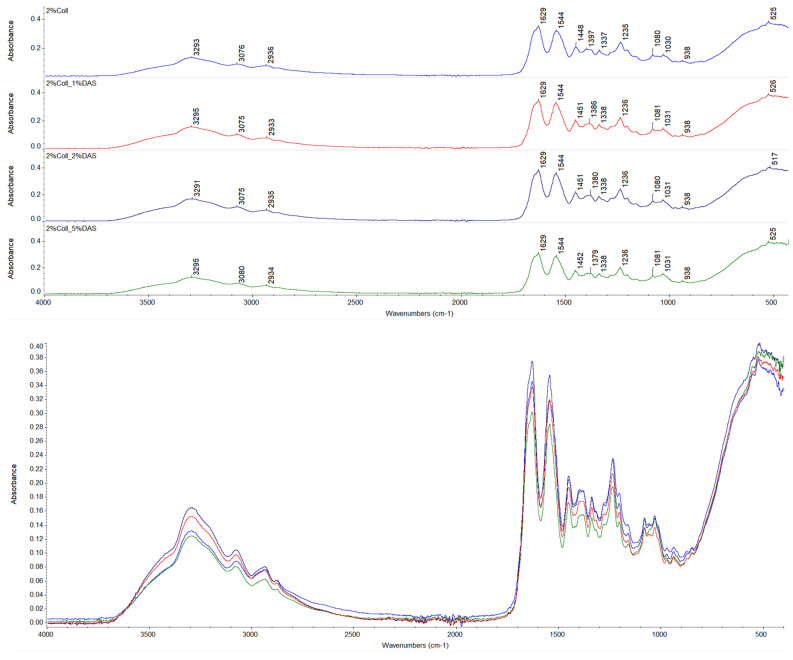
FTIR spectra for collagen films obtained from 2% solution with DAS addition (experiment conditions: resolution = 4 cm^−1^; wavenumber range = 400–4000 cm^−1^; 64 scans).

The chemical structure of prepared collagen films was investigated by FTIR spectroscopy. Spectra of native collagen films and collagen films with DAS addition are presented in Figure 1 and Figure 2, respectively. The following characteristic bands for collagen were found on the spectra: Amide A bands at about 3300 cm^−1^, Amide B bands at about 3080 cm^−1^, asymmetrical stretch of –CH_2_ bands at about 2930 cm^−1^, Amide I at about 1630 cm^−1^, Amide II at about 1540 cm^−1^, bend of –CH_2_ bands at about 1450 cm^−1^, symmetrical stretch of COO– at 1379–1397 cm^−1^, bands corresponding to in-plane OH (phenol) bending at 1337–1338 cm^−1^, Amide III band at 1235–1237 cm^−1^, and stretch of C–O and asymmetrical and symmetrical stretch of C–O–C bands at 1080 and 1030 cm^−1^, respectively [34,35,36,37]. Wavenumbers for the mentioned IR characteristic bands for collagen samples are presented in Table 2. For non-cross-linked and DAS-cross-linked collagen samples, wavenumbers for characteristic bands remained almost at the same positions; thus, it might indicate that the collagen secondary structure was not influenced; however, changes can be observed in characteristic band intensities [38]. Based on the assessment of the Amide I band, which allows for protein secondary structure analysis [39,40,41], changes in its intensity for cross-linked samples might reflect created interactions between a cross-linking agent and the collagen matrix [38]. Moreover, it can be also observed that all 1% and 2% collagen films with cross-linking agents showed alternations of the band corresponding to a symmetrical stretch of COO– to lower wavenumbers, which also might be associated with carboxyl groups’ involvement in new interactions. Furthermore, only 5% DAS addition to 1% collagen films caused a small shift of the Amide II band (which provides information regarding the conformation of proteins) to a higher wavenumber [41]. Moreover, it can be noticed that the increased intensity of the band at 1030 cm^−1^ might be correlated with DAS compound addition (the highest intensity was for 5% DAS addition), which is a characteristic band for dialdehyde starch corresponding to C–O stretching vibrations [41], while for 2% collagen samples with 5% DAS addition, bands at 1030 cm^−1^ presented slightly different shapes and were wider.

### 3.2. Mechanical Analysis

**Figure 3:**
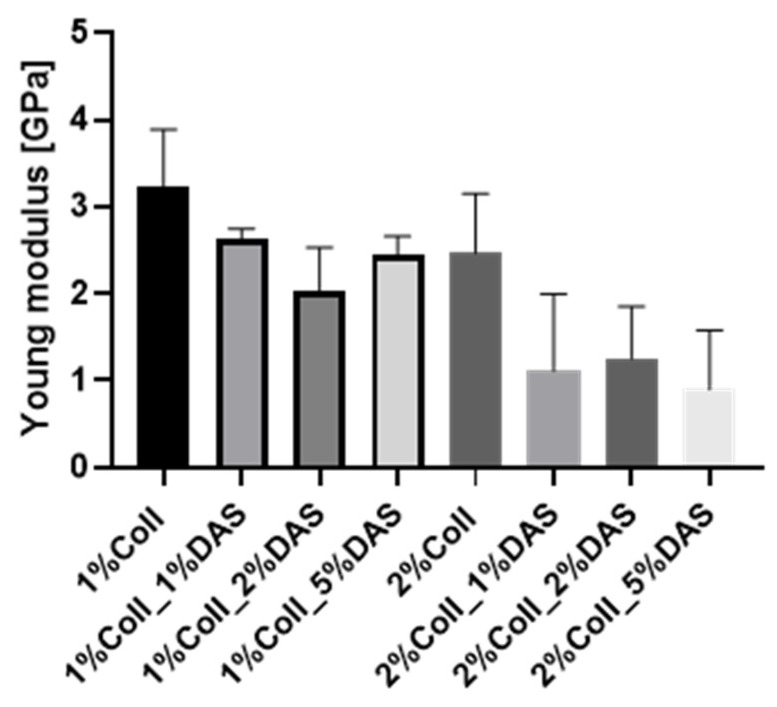
Young Modulus for collagen films with DAS addition (experiment conditions: the speed starting position equaled 50 mm/min, the speed of the initial force 5 mm/min, and the initial force 0.1 MPa).

**Figure 4:**
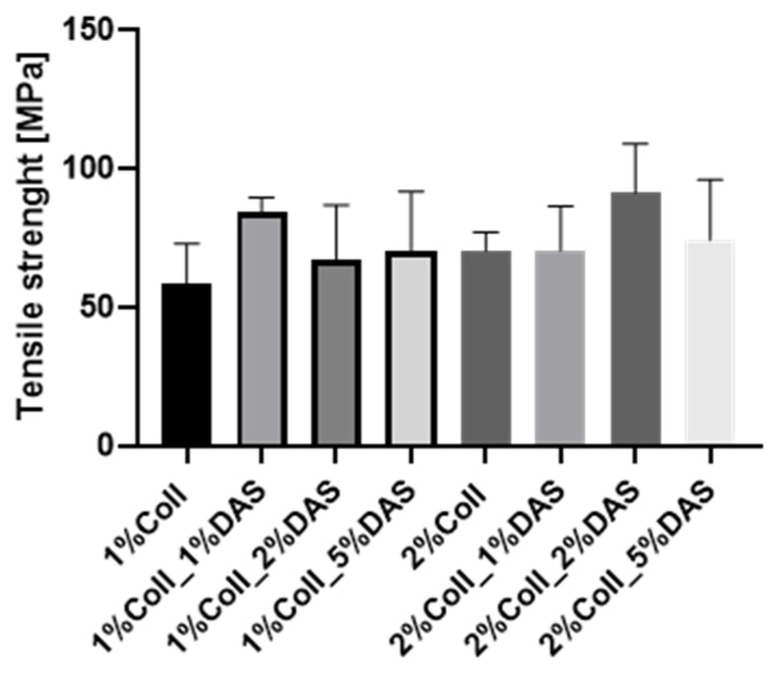
Tensile strength for collagen films with DAS addition (experiment conditions: the speed starting position equaled 50 mm/min, the speed of the initial force 5 mm/min, and the initial force 0.1 MPa).

**Figure 5:**
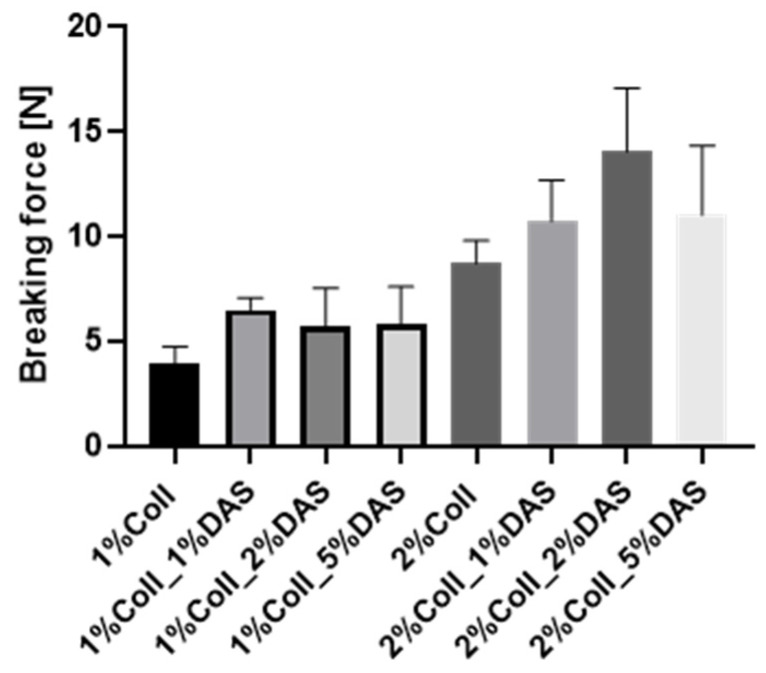
Breaking force for collagen films with DAS addition (experiment conditions: the speed starting position equaled 50 mm/min, the speed of the initial force 5 mm/min, and the initial force 0.1 MPa).

**Figure 6:**
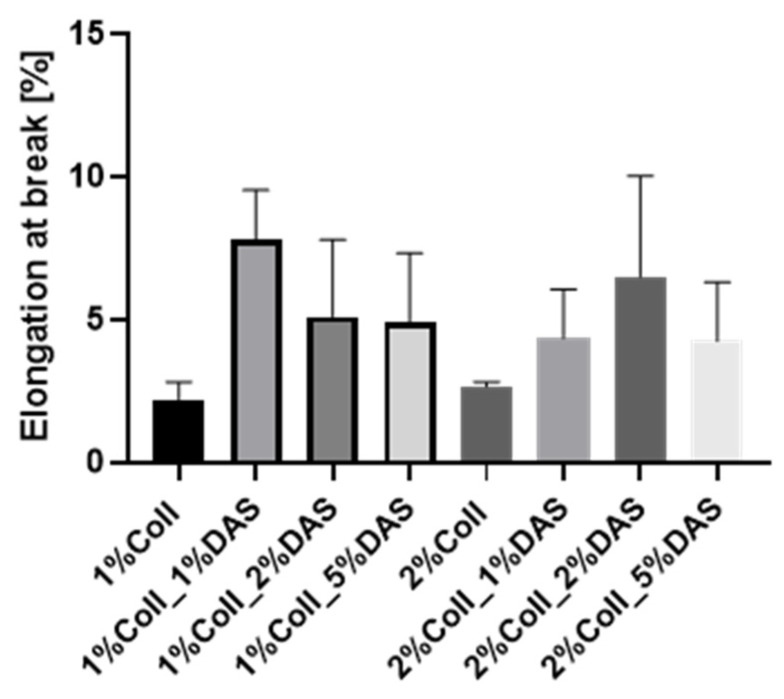
Elongation at break for collagen films with DAS addition (experiment conditions: the speed starting position equaled 50 mm/min, the speed of the initial force 5 mm/min, and the initial force 0.1 MPa).

DAS addition to collagen films influenced the mechanical parameters of collagen films. Results for the Young’s Modulus, tensile strength, breaking force, and elongation at break parameters are presented in Figure 3, Figure 4, Figure 5 and Figure 6. Mechanical testing indicated that DAS incorporation into collagen films caused a decrease in the Young’s Modulus parameter and the lower values were observed for all 2% collagen film samples with a cross-linking agent. Moreover, increased tensile strength values were observed for samples 1%Coll_1%DAS and 2%Coll_2%DAS. Higher values of breaking force parameters were obtained for DAS-modified films; furthermore, 2% collagen samples indicate higher values than 1% with the highest value obtained for the 2%Coll_2%DAS sample. The addition of a cross-linking agent resulted in higher values of the elongation at break parameter; again, the highest values were observed for 1%Coll_1%DAS and 2%Coll_2%DAS, which indicate that these samples were the most flexible.

### 3.3. Contact Angle and Surface Energy

The results for contact angle analysis are presented in Table 3. Images of drops during contact angle measurements for collagen films with DAS addition are presented in Table 4. The cross-linking agent had an effect on the contact angle of collagen films. The contact angle was significantly higher for modified films than unmodified ones. DAS addition to collagen films caused a decrease in the hydrophilicity and surface free energy. In particular, the calculated polar surface energy might suggest a more hydrophobic character of obtained samples and their lower wettability.

### 3.4. Swelling and Degradation Analysis

**Figure 7:**
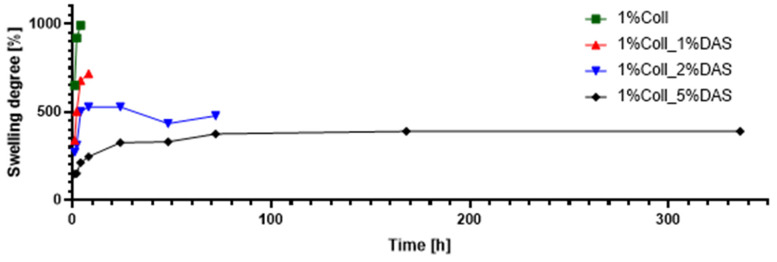
Swelling degree for collagen films (1%) with DAS addition (experiment conditions: swelling in PBS at 37 °C).

**Figure 8:**
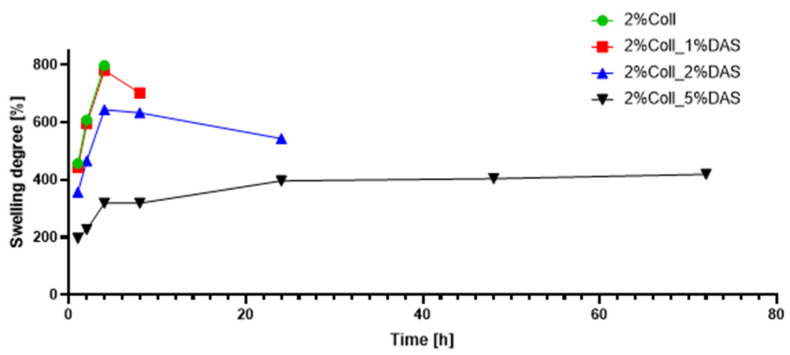
Swelling degree for collagen films (2%) with DAS addition (experiment conditions: swelling in PBS at 37 °C).

Results of swelling analysis for both 1% and 2% collagen films modified with DAS are presented in Figure 7 and Figure 8. Both pure collagen films fell apart after four hours of immersion in PBS solution. The addition of DAS had an impact on the swelling degree and stability of obtained materials, as one can observe that DAS incorporated into collagen films reduced swelling degree for all samples in comparison to native collagen film, meaning that samples with DAS immersed in PBS solution were more stable for a longer time. Analysis showed that the higher the DAS content in collagen films, the lower the degree of swelling and the slower the film degradation. The lowest swelling degree and the slowest degradation were observed for both samples with the highest addition of DAS, i.e., 5%. For 2% collagen with 5% DAS addition, after three days, degradation of the sample was observed, while for 1% collagen with 5% DAS addition, the same was observed after fourteen days.

### 3.5. Atomic Force Microscopy (AFM)

Surface morphology of non-cross-linked and DAS-cross-linked collagen samples was investigated with Atomic Force Microscopy. Roughness values are presented in Table 5. AFM images of collagen films with additives are presented in Table 6. There were slight differences in roughness parameters between collagen films. However, the lowest values of the Rq parameter were noticed for 2% collagen films with 2% and 5% DAS addition and were approximately 10 nm, while the highest were observed for 1% collagen films and 1% collagen samples with 1% DAS addition (16.79 and 17.92, respectively); thus, the latter were characterized by the highest roughness. The 1% collagen films presented higher roughness than 2% collagen films; it can be observed that cross-linking agent addition modified collagen film surface morphology: 1% addition of DAS in both cases caused an increase in the Rq parameter, while 2% and 5% DAS addition caused lower roughness of materials.

### 3.6. Thermogravimetric Analysis

**Figure 9:**
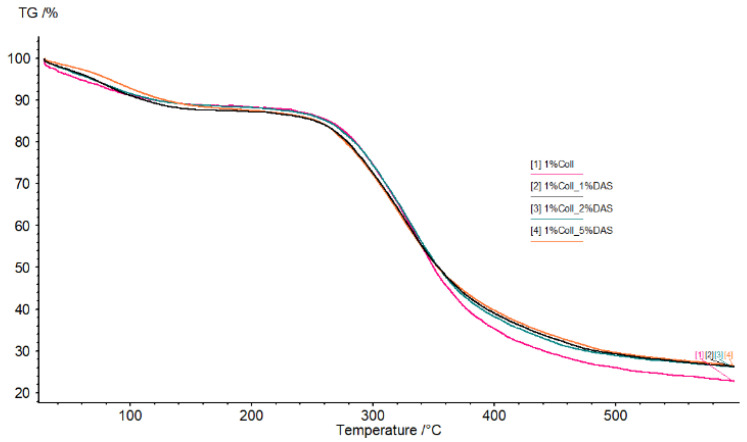

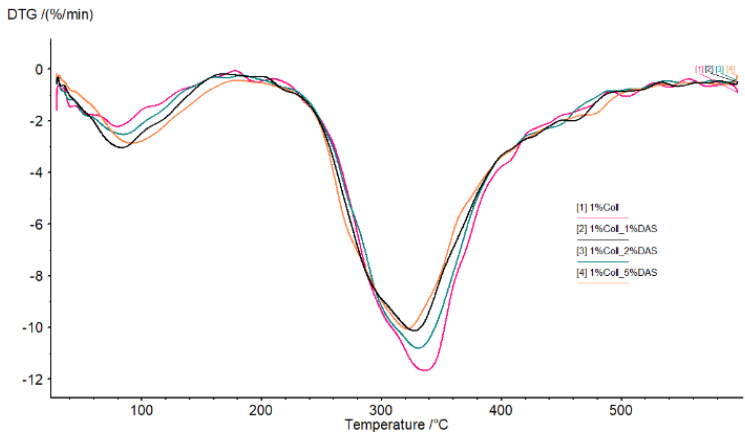
TG and DTG curves obtained for unmodified and modified collagen films (obtained from 1% solution) (temperature range from 20 to 600 °C at a heating rate of 20 °C/min in nitrogen atmosphere).

**Figure 10:**
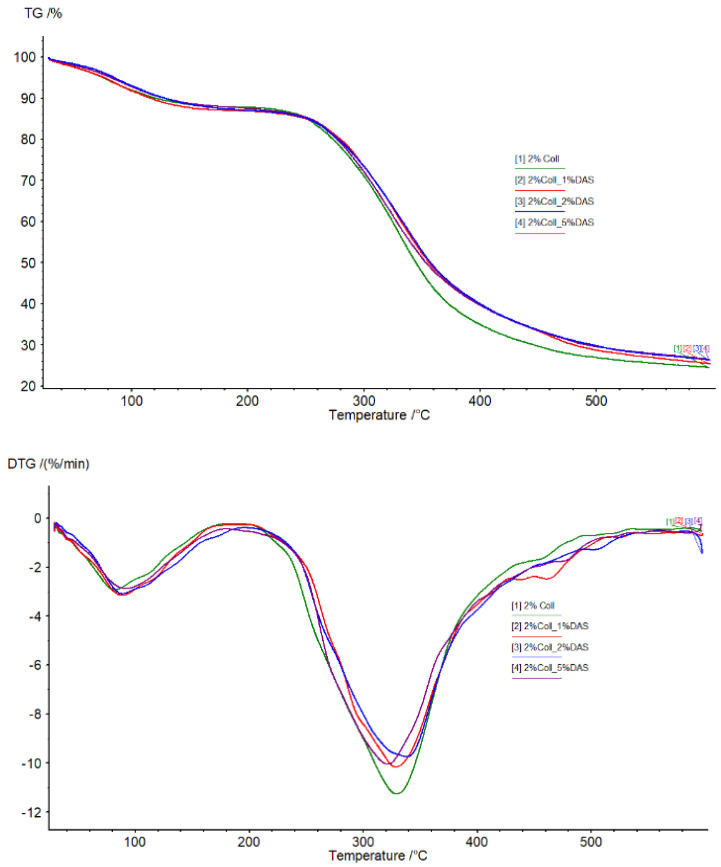
TG and DTG curves obtained for unmodified and modified collagen films (obtained from 2% solution) (temperature range from 20 to 600 °C at a heating rate of 20 °C/min in nitrogen atmosphere).

TG and DTG curves of collagen films modified with DAS are shown in Figure 9 and Figure 10. Analyzing thermograms for all samples, the first region was probably related to water molecules’ elimination from the films, while the second region was related to material degradation. There were no significant differences in thermograms for all obtained samples; however, it can be noticed that the pure collagen sample was less thermally stable than the cross-linked collagen samples.

## 4. Discussion

Research has shown that dialdehyde starch applied as a cross-linking agent influences collagen film properties. Prepared films were smooth and homogenous.

### 4.1. FTIR Spectroscopy

Registered FTIR spectra presented characteristic bands for collagen. There were no significant changes in wavenumbers of characteristic bands of collagen samples after cross-linking, which might indicate that the collagen secondary structure was not affected; however, changes could be observed in characteristic band intensities. Alterations of a band corresponding to a symmetrical stretch of COO− were observed. This might be related to interactions between the cross-linking agent and collagen. DAS’s mechanism of cross-linking is related to the reaction of the aldehyde groups of DAS with amine groups on the collagen fibril surface (Schiff base formation) [32]. Liu et al. noticed that DAS concentrations from 0 to 0.0111 mg mL^−1^ significantly increased the cross-linking degree, which corresponds to a DAS/collagen weight ratio of 1:120. This DAS concentration was a limit value, above which dialdehyde starch concentration did not affect the cross-linking level [32].

### 4.2. Mechanical Testing

The cross-linking process influenced the mechanical parameters of collagen films. After mechanical testing, it was noticed that DAS incorporation into collagen films caused a decrease in Young’s Modulus and an increase in breaking force and elongation at break parameters, which might be associated with higher flexibility of samples. Tensile strength was the highest for the 1% collagen film with 1% cross-linking agent addition and for the 2% collagen sample with 2% DAS addition. Węgrzynowska-Drzymalska et al. examined the influence of dialdehyde starch nanocrystals on chitosan, gelatin, and a mixture of these two natural polymers’ mechanical properties. The tensile strength values were higher for all samples modified with DAS, which partially corresponds to our research [42]. Tang et al. found that after DAS addition to chitosan, the tensile strength increased with the increase in the DAS content, and a similar tendency has been observed for elongation at break [43]. Spence et al. showed that materials made of zein and dialdehyde starch presented different mechanical properties depending on the oxidation degree. It was observed that the increased oxidation degree of starch contributed to increase the tensile strength, Young’s modulus, and percentage elongation of obtained materials [44]. Research by Bajer et al. indicated that composite materials made of native starch and dialdehyde starch presented lower stress at break and elastic moduli and higher elongation at break in comparison to materials made of starch, indicating greater flexibility of obtained films with DAS addition caused by created interaction between dialdehyde starch, starch, and glycerol [45].

### 4.3. Contact Angle and Surface Energy

The results for contact angle were significantly higher for collagen films cross-linked with DAS; thus, samples were characterized by lower hydrophilicity or a more hydrophobic character and lower wettability. Selected physicochemical properties of gelatin/DAS films were evaluated by Nguyet et al. They also indicated that the higher the aldehyde content in films modified with DAS, the lower the hydrophilicity of the samples [46]. Bajer et al. conducted contact angle measurements of native starch and dialdehyde starch blends, which resulted in confirmation of the hydrophobic influence of dialdehyde starch on studied materials [45].

### 4.4. Swelling and Degradation Analysis

Modified samples presented a lower swelling degree in PBS solution than native collagen films, which degraded after four hours of immersion in PBS solution. The higher the DAS concentration in collagen films, the lower the swelling degree and the slower the film degradation. The lowest one was observed for 1 and 2% collagen samples with 5% DAS addition, for which degradation of the samples was observed after 14 and 3 days, respectively. Tang et al. examined the swelling properties of chitosan films modified with DAS. Studies indicated that the swelling degree of cross-linked samples rapidly increased with the increase in additive content. An addition of DAS equaling 5% was a significant value; after achieving this content, the degree of swelling was decreased. The higher the DAS content in the material, the greater the cross-linking degree of chitosan, which makes it more difficult for water to permeate the film [43]. Moreover, Valipour et al. indicated that dialdehyde starch content had an impact on hydrogels made of collagen and chitosan; thus, the lower content of cross-linking agent applied in this study contributed to the highest swelling ratio and biodegradability [22]. Carrera et al.’s studies on hydrogels based on polyvinyl alcohol with the addition of dialdehyde starch resulted in the evaluation of the swelling process of the proposed material. Various levels of DAS oxidation give different effects. Hydrogels with low- and medium-oxidation DAS exhibited higher water absorption capacity but lower stability in time, which remains contrary to hydrogels containing high-oxidation DAS [47]. Studies have shown that the oxidation degree of starch in materials made of zein and dialdehyde starch also influences water absorption; thus, increased oxidation degree causes a decrease in water absorption of materials. Studies indicated that samples made of zein and polymeric dialdehyde starch (90% oxidized starch) after immersion in deionized water did not indicate swelling, which is an important feature for materials applied in environments characterized by high humidity [44]. Mechanical parameters and swelling degree are very important in terms of creating wound dressings, successfully accelerating the healing of skin damage, ensuring adequate strength of the material, and effectively managing wound exudate.

### 4.5. Atomic Force Microscopy (AFM)

Based on AFM images and roughness values, it was noticed that DAS modified collagen film surface morphology. It was found that a 1% addition of DAS in both collagen film concentrations caused an increase in roughness, while 2% and 5% DAS addition caused a decrease. The tendency of roughness values of films with DAS observed in this study is different from that observed by Oluwasina et al. [48]. Various results may be caused by the type of modifying material; different materials have various levels of compatibility with dialdehyde starch. Low values of roughness may indicate good cross-linking level and miscibility [48].

### 4.6. Thermogravimetric Analysis

Analyzing thermograms for collagen samples, there were no significant differences; however, it can be noticed that pure collagen samples were less thermally stable than cross-linked ones. Some reports confirm dialdehyde starch’s ability to increase the thermal resistance of materials. Oluwasina et al. obtained bioplastic films consisting of starch, silica, and dialdehyde starch. Materials containing DAS showed higher temperatures of degradation compared to films without this additive [48]. Moreover, improved thermal stability in comparison to starch and dialdehyde starch indicated dialdehyde starch nanoparticles according to research conducted by Yu et al. [49]. Furthermore, as Zhang et al. showed in their studies, dialdehyde starch can be modified with methanol, ethanol, or glycol, resulting in higher thermal stability, which is important in terms of mechanical properties [50].

According to the scientific literature, our studies fit into current research and the expectations to create new biopolymer-based materials for skin regeneration. Our research is in line with the principles of sustainability by using biodegradable and environmentally friendly materials. The collagen used in this study was obtained from fish skins, which are often a waste product of fish processing [7]. The method presented for obtaining biopolymer films is relatively simple and does not require large quantities of reagents. The results of our experiments are innovative and constitute the next step in understanding and determining the physicochemical properties of collagen and dialdehyde starch blends. The conducted research expands the state of knowledge about these polymers, which are important from a biomedical and cosmetological point of view and constitute a contribution to further research, which still in the context of collagen and dialdehyde starch remains scarce.

## 5. Conclusions

Dialdehyde starch is a promising cross-linking agent for collagen extracted from fish skin. It improves collagen’s mechanical properties and its thermal stability. The swelling properties of collagen films can be modified by the amount of added dialdehyde starch. Based on the research conducted, certain conclusions can be drawn. Research has shown that dialdehyde starch incorporation into collagen films influences their properties. FTIR spectra indicated that after cross-linking, there were no significant changes in wavenumbers of characteristic bands of collagen; however, changes could be observed in characteristic band intensities. Based on mechanical testing results, DAS caused a decrease in Young’s Modulus and an increase in breaking force and elongation at break parameters, which might be associated with the higher flexibility of samples. Obtained results for contact angle indicated that samples with DAS addition were characterized by lower hydrophilicity and wettability than pure collagen films. AFM analysis suggested that DAS modified collagen film surface morphology; it was found that a 1% addition of DAS in both collagen film concentrations caused an increase in roughness, while 2% and 5% DAS addition caused a decrease. It was also observed that native collagen samples were less thermally stable than cross-linked ones. Moreover, it was noticed that the higher the DAS concentration in collagen films, the lower the swelling degree and the slower the film degradation. Both collagen and dialdehyde starch, due to their safety and non-toxicity, are desirable compounds in the design of wound dressings based on natural polymers and other biomedical and cosmetic applications. However, further studies such as antimicrobial activity measurements or cytotoxicity assessments are essential to confirm their beneficial impact on the skin and usefulness in wound dressings.

## Figures and Tables

**Table 1:** Photos of prepared collagen films (from 1 and 2% *w*/*w* solutions) with DAS addition (1, 2 and 5%, respectively).

1% Coll 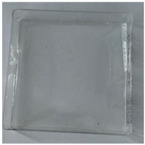	2% Coll 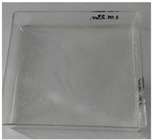
1%Coll_1%DAS 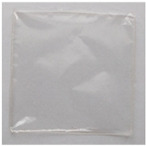	2%Coll_1%DAS 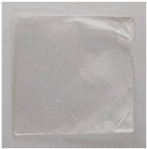
1% Coll_2%DAS 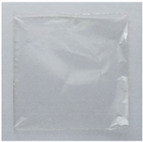	2%Coll_2%DAS 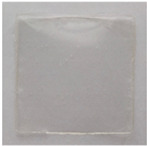
1%Coll_5%DAS 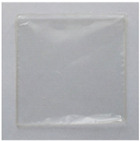	2% Coll _5%DAS 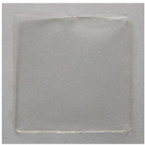

**Table 2:** Wavenumbers of characteristic bands for collagen films.

Characteristic Bands [cm^−1^]	Amide A	Amide B	CH_2_ Asymmetric Stretch	Amide I	Amide II	CH_2_ Bend	COO^−^ Symmetric Stretch	in-Plane OH (Phenol) Bending	Amide III	C–O Stretch/ C–O–C Asymmetric Stretch	C–O Stretch/ C–O–C Symmetric Stretch
1%Coll	3297	3077	2931	1632	1541	1449	1397	1338	1235	1080	1030
1%Coll_1%DAS	3295	3080	2933	1629	1541	1451	1382	1337	1236	1080	1031
1%Coll_2%DAS	3296	3078	2934	1629	1544	1451	1385	1337	1237	1080	1031
1%Coll_5%DAS	3296	3081	2932	1629	1548	1452	1386	1337	1237	1080	1031
2%Coll	3293	3076	2936	1629	1544	1448	1397	1337	1235	1080	1030
2%Coll_1%DAS	3295	3075	2933	1629	1544	1451	1386	1338	1236	1081	1031
2%Coll_2%DAS	3291	3075	2935	1629	1544	1451	1380	1338	1236	1080	1031
2%Coll_5%DAS	3295	3080	2934	1629	1544	1452	1379	1338	1236	1081	1031

**Table 3:** Contact angle and surface energy results for collagen films with DAS addition.

Sample	Θ Glycerine [°]	Θ Diodomethane [°]	IFT (s) [mJ/m^2^]	IFT (s, D) [mJ/m^2^]	IFT (s, P) [mJ/m^2^]
1%Coll	58.7	36.8	44.01	33.33	10.68
1%Coll_1%DAS	90.4	39.9	40.38	40.29	0.09
1%Coll_2%DAS	94.4	40.8	40.95	40.94	0.01
1%Coll_5%DAS	86.4	45.3	36.5	35.69	0.81
2%Coll	61.3	38.5	42.62	33.04	9.58
2%Coll_1%DAS	91.1	48.5	35.21	34.91	0.30
2%Coll_2%DAS	102.3	46.4	40.03	39.58	0.44
2%Coll_5%DAS	88.6	45.8	36.48	35.99	0.49

**Table 4:** Images of drop (G—glycerine, D—diodomethane) during contact angle measurements for collagen films with DAS addition.

	**1%Coll**	**1%Coll_1%DAS**	**1%Coll_2%DAS**	**1%Coll_5%DAS**
G	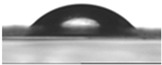	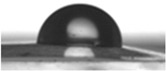	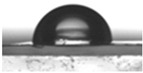	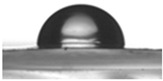
D	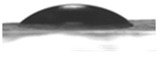	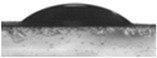	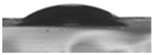	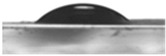
	**2%Coll**	**2%Coll_1%DAS**	**2%Coll_2%DAS**	**2%Coll_5%DAS**
G	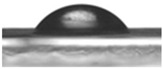	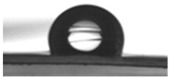	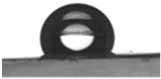	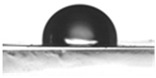
D	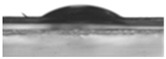	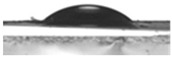	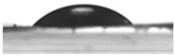	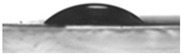

**Table 5:** Rq and Ra values for collagen films with DAS addition (scanned area 5 μm × 5 μm).

	R_q_ [nm]	R_a_ [nm]
1%Coll	16.79 ± 0.50	13.57 ± 0.75
1%Coll_1%DAS	17.92 ± 3.61	14.46 ± 2.93
1%Coll_2%DAS	11.98 ± 2.84	9.65 ± 2.15
1%Coll_5%DAS	13.70 ± 2.81	11.09 ± 2.06
2%Coll	13.45 ± 3.25	10.92 ± 2.55
2%Coll_1%DAS	15.80 ± 0.18	12.51 ± 0.04
2%Coll_2%DAS	10.31 ± 2.91	8.25 ± 2.29
2%Coll_5%DAS	10.65 ± 3.04	8.60 ± 2.53

**Table 6:** AFM images of the surface of collagen film with DAS (scanned area: 5 μm × 5 μm).

1% Coll 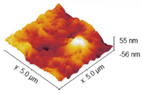	2% Coll 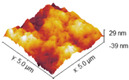
1%Coll_1%DAS 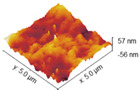	2%Coll_1%DAS 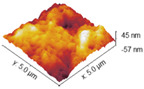
1% Coll_2%DAS 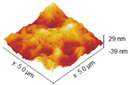	2%Coll_2%DAS 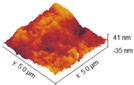
1%Coll_5%DAS 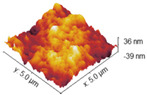	2% Coll _5%DAS 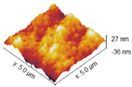

## Data Availability

Data are protected by the company.
